# Integrating Next-Generation Reproductive Organoids with Genomics, Multi-Omics and Bioengineering to Understand Human Infertility

**DOI:** 10.3390/genes17091080

**Published:** 2026-09-08

**Authors:** Paul Barreau, Cyril Djari, John De Vos, Nino-Guy Cassuto, Said Assou

**Affiliations:** 1 Institute for Regenerative Medicine and Biotherapy (IRMB), University of Montpellier, INSERM, CHU Montpellier, 34295 Montpellier, France; paul.barreau@etu.umontpellier.fr (P.B.); john.devos@inserm.fr (J.D.V.); 2 Department of Genetic Medicine and Development, University of Geneva, 1211 Geneva, Switzerland; cyril.djari@unige.ch; 3 ART Unit, Drouot Laboratory, 75009 Paris, France; guycassuto@labodrouot.com

**Keywords:** genomics, multi-omics, organoid, infertility

## Abstract

Infertility affects approximately one in six individuals worldwide and is a highly heterogeneous disorder resulting from the complex interplay of genetic, epigenetic, endocrine, environmental and lifestyle factors. Although numerous genes and molecular pathways involved in female and male infertility have been identified, elucidating the functional consequences of disease-associated variants remains challenging due to the lack of relevant human experimental models. In recent years, reproductive organoids have emerged as powerful three-dimensional systems that recapitulate key structural, cellular and functional characteristics of the ovary, fallopian tube, endometrium, testis and early embryo. Here, we review the current landscape of reproductive organoid models based on a focused analysis of the literature, with particular emphasis on original studies describing their generation, characterization and applications. Beyond modeling tissue development and reproductive physiology, these models provide unique opportunities to investigate infertility-associated mechanisms, gene regulatory networks, cell–cell communication and tissue-specific responses to environmental and pharmacological stimuli. Single-cell and spatial transcriptomics, multi-omics, CRISPR/Cas9 genome editing, artificial intelligence and bioengineering technologies, including organ-on-chip systems, are expanding their potential as next-generation platforms for functional genomics, disease modeling, biomarker discovery and therapeutic screening. However, current reproductive organoids remain simplified representations of native tissues, with limitations in physiological maturity, reproducibility and standardization, while their clinical predictive value remains to be established. Overall, by linking genomic variation with molecular regulation, cellular phenotypes and tissue organization, reproductive organoids represent promising preclinical platforms for understanding human infertility and may ultimately contribute to the development of precision reproductive medicine.

## 1. Introduction

Infertility affects approximately one in six individuals worldwide, with a pooled lifetime prevalence estimated at 17.5% [1]. Clinically, infertility is defined as the inability to achieve pregnancy after 12 months of regular unprotected sexual intercourse [2]. Human fertility relies on the coordinated function of the reproductive organs and on complex interactions between genetic, epigenetic, endocrine, immunological, environmental and lifestyle factors [3]. Accordingly, infertility is a highly heterogeneous condition that may involve one or both partners. Female infertility may result from diminished ovarian reserve, ovulatory dysfunction, tubal abnormalities, endometriosis or impaired endometrial receptivity [4,5], whereas male infertility is commonly associated with defective spermatogenesis, endocrine dysfunction or reproductive tract abnormalities [6,7]. Advanced parental age may further compromise reproductive potential through progressive depletion of the ovarian reserve and deterioration of oocyte quality in women [8,9] and age-related alterations in reproductive function and sperm quality in men [10]. Environmental and lifestyle factors can additionally interact with individual susceptibility and epigenetic regulation to influence reproductive function [3]. Nevertheless, approximately 15–30% of infertility cases remain unexplained, depending on the diagnostic criteria and population investigated [11]. Beyond its biological determinants, infertility also imposes a substantial psychological and psychosocial burden on both women and men, with emotional distress, anxiety and depressive symptoms frequently reported during infertility investigation and treatment [12].

Advances in genomics have considerably expanded our understanding of the molecular basis of human infertility, revealing contributions from monogenic defects, chromosomal abnormalities, polygenic susceptibility, epigenetic dysregulation and environmental influences [3,6]. Large-scale sequencing studies have identified numerous genes involved in gonadal development, meiosis, DNA repair, hormone signaling and germ cell differentiation [6,13,14]. In women, pathogenic variants affecting folliculogenesis, oocyte maturation and meiotic progression have been associated with primary ovarian insufficiency (POI), while chromosomal abnormalities such as Turner syndrome remain important causes of ovarian dysfunction [13,15,16]. Genetic susceptibility also contributes to complex reproductive disorders, including endometriosis, implantation failure and recurrent pregnancy loss [17,18,19,20]. In men, Y-chromosome AZF microdeletions, chromosomal abnormalities such as Klinefelter syndrome and pathogenic variants affecting meiosis and spermatogenesis are established genetic causes of severe infertility [6,21,22,23]. In parallel, epigenetic mechanisms, including DNA methylation, histone modifications, chromatin remodeling and non-coding RNAs, contribute to the regulation of gametogenesis, fertilization, embryo development and implantation and may mediate interactions between genetic and environmental factors [3].

Despite these advances, translating genomic discoveries into biological mechanisms remains challenging because the functional consequences of many infertility-associated variants cannot be adequately investigated using conventional experimental models. Animal models are limited by species-specific differences, whereas two-dimensional (2D) cell cultures fail to reproduce the three-dimensional architecture, cellular heterogeneity and dynamic microenvironment of human reproductive tissues [24,25]. Reproductive organoids have therefore emerged as physiologically relevant 3D models capable of recapitulating key structural, molecular and functional features of reproductive tissues. Derived from primary tissues, adult stem cells or pluripotent stem cells, these models preserve important aspects of tissue architecture and multicellular organization and provide new opportunities to investigate reproductive development, tissue homeostasis and infertility-associated mechanisms [24,25,26,27,28,29]. Their combination with patient-derived induced pluripotent stem cells (iPSCs), genome editing, single-cell and spatial omics, multi-omics, artificial intelligence and organ-on-chip technologies further expands their potential for functional studies of reproductive disorders.

In this review, we summarize recent advances in ovarian, fallopian tube, endometrial, testicular and embryo-like organoid models and discuss their contribution to understanding female and male infertility. Particular emphasis is placed on their integration with genomics, multi-omics, genome engineering and bioengineering approaches for functional genomics, disease modeling and drug discovery, as well as their potential future contribution to precision reproductive medicine.

### Literature Search Strategy

This review was based on a focused literature search conducted using PubMed, Web of Science and Google Scholar. Searches combined terms related to reproductive organoids and infertility with terms including ovary, fallopian tube, endometrium, testis, embryo-like models, genomics, single-cell and spatial omics, multi-omics, genome editing, organ-on-chip technologies and artificial intelligence. Particular attention was given to original research articles describing the generation, characterization and experimental applications of reproductive organoid models. Relevant review articles and key publications were additionally considered to provide broader biological and clinical context.

## 2. Female Reproductive Tract Organoids for Infertility Research

### 2.1. Overview

Female fertility depends on the coordinated function of the ovaries, fallopian tubes and endometrium that are sequentially implicated in ovulation, fertilization, embryo transport and implantation. Ovaries produce developmentally competent metaphase II oocytes. The fallopian tubes provide the optimal microenvironment for fertilization and support early embryonic development until the blastocyst reaches the uterine cavity [30]. The endometrium undergoes cyclic remodeling to establish the receptive state required for embryo implantation and placenta development. Consequently, dysfunctions of any of these reproductive compartments (e.g., ovarian insufficiency, tubal disease/dysfunction or impaired endometrial receptivity) may compromise the reproductive success and result in infertility. Female factors alone account for ~20% of infertility cases, although they frequently coexist with male or combined etiologies [31].

### 2.2. Ovary and Ovarian Organoids

The ovary is the primary female reproductive organ, fulfilling both endocrine and gametogenic functions through steroid hormone production and the cyclic maturation of oocytes. The female reproductive lifespan depends on a finite ovarian reserve that is established during fetal development and that progressively declines because of physiological follicular atresia, resulting in an age-related reduction in oocyte quantity and quality [32,33,34]. Ovarian dysfunction is one of the leading causes of female infertility. For instance, POI, defined as the loss of ovarian function before the age of 40 years, is characterized by hypoestrogenism, elevated gonadotropin levels and impaired folliculogenesis. Its etiology is highly heterogeneous and includes chromosomal abnormalities, pathogenic gene variants that affect ovarian development, folliculogenesis and meiosis, autoimmune disorders, infections, as well as iatrogenic damage induced by chemotherapy, radiotherapy or ovarian surgery [14,35]. Despite considerable advances in reproductive medicine, the molecular mechanisms that regulate follicular activation, ovarian aging and POI remain incompletely understood, largely because 2D cell cultures and animal models do not fully reproduce the complex cellular interactions in the human ovary. To overcome these limitations, ovarian organoids have emerged as physiologically relevant 3D models that faithfully recapitulate key features of the ovarian architecture, folliculogenesis, steroidogenesis and cell–cell communication. Compared with conventional in vitro systems, these models better preserve the structural and functional properties of ovarian tissue, providing valuable platforms to study ovarian development, endocrine regulation and infertility-associated disorders. One of the most significant advances has been the generation of human follicle-like organoids (ovaroids) derived from iPSCs. Through the overexpression of *NR5A1* and *RUNX1/2*, human iPSCs can be differentiated into granulosa-like cells that express canonical granulosa markers, including *AMH*, *FOXL2* and *CYP19A1* [36]. When co-cultured with human primordial germ cells in 3D conditions, these granulosa-like cells self-organize into follicle-like structures that closely resemble native ovarian follicles and exhibit functional steroidogenesis through FSH-responsive estradiol secretion (Table 1).

By reproducing essential features of early folliculogenesis, these ovaroids provide unprecedented opportunities to investigate ovarian development, follicular maturation and the mechanisms underlying ovarian disorders, particularly POI. Besides disease modeling, ovarian organoids are increasingly recognized as promising tools for regenerative and precision reproductive medicine. In a proof-of-concept study, bioengineered ovarian constructs composed of granulosa and theca cells encapsulated within alginate restored endocrine function following transplantation in ovariectomized rats. They maintained physiological hormone production for ~90 days and corrected endocrine deficiencies associated with ovarian failure [37]. Ovarian organoids reproduce the ovarian architecture and also allow the mechanistic investigation of the molecular events underlying follicular activation, granulosa–oocyte communication and ovarian aging. Unlike 2D cell culture systems, these models preserve the dynamic interactions between germ cells, granulosa cells and the surrounding microenvironment that govern folliculogenesis and endocrine function. Their integration with patient-derived iPSCs, CRISPR/Cas9 genome editing and single-cell multi-omics provides a framework for the functional investigation of disease-causing gene variants associated with POI (e.g., *FOXL2*, *BMP15*, *GDF9*, *SOHLH1*, *MCM8*, *MCM9* and *NR5A1*) [13,15,44]. However, direct organoid-based functional validation has not yet been established for all these genes, and for several of them, this remains a prospective experimental strategy rather than a routinely demonstrated approach.

**Figure 1:**
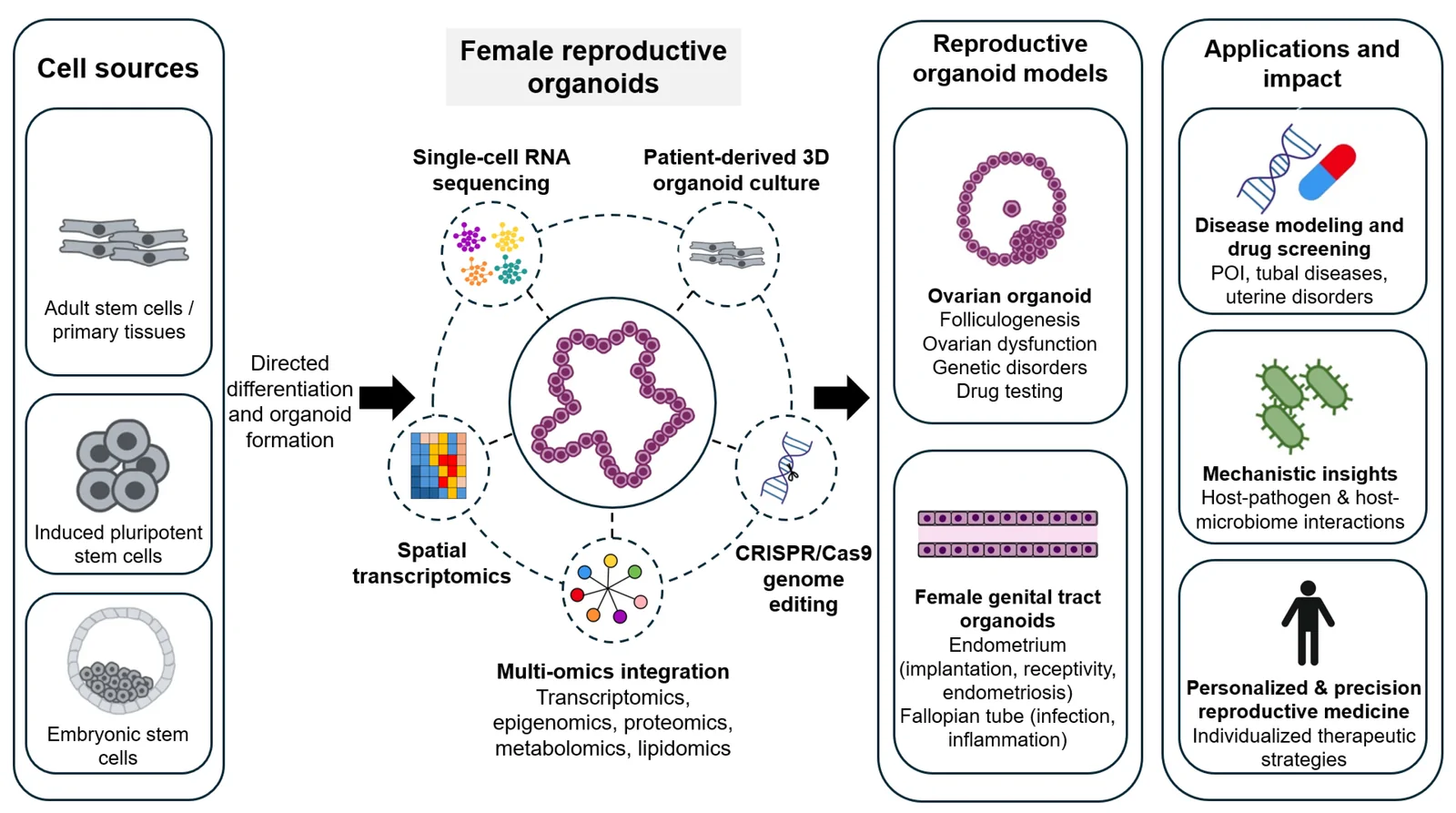
Next-generation female reproductive organoids that integrate genomics, multi-omics and bioengineering to study female infertility. Female reproductive organoids are generated from adult stem cells, primary tissues, induced pluripotent stem cells, or embryonic stem cells. The integration of patient-derived organoids with single-cell RNA sequencing, spatial transcriptomics, CRISPR/Cas9 genome editing and multi-omics allows the high-resolution investigation of ovarian and female genital tract biology. These models support disease modeling, mechanistic studies and preclinical drug screening, while offering future perspectives for precision reproductive medicine.

By linking genetic variation with transcriptional programs, cellular phenotypes and hormone production, ovarian organoids provide an unprecedented experimental framework for deciphering disease mechanisms, validating candidate infertility genes and identifying novel therapeutic targets. Consequently, these models are evolving from descriptive culture systems toward functional genomic platforms that may ultimately contribute to precision reproductive medicine, although such applications remain preclinical (Figure 1).

### 2.3. Fallopian Tube Organoids

Fallopian tubes are an essential component of the female reproductive tract, providing the physiological environment for oocyte capture, sperm transport, fertilization and early preimplantation embryo development. These functions depend on the coordinated activity of secretory and ciliated epithelial cells and smooth muscle contraction to regulate the transport of gametes and embryos through the tubal lumen [45]. Structural and functional alterations of the fallopian tubes are one of the leading causes of female infertility. Tubal factor infertility is most commonly associated with partial or complete tube obstruction, preventing fertilization or impairing embryo transport to the uterine cavity [46]. Chronic salpingitis caused by *Chlamydia trachomatis* remains the principal cause of tube damage. As these infections are frequently asymptomatic, persistent inflammation can induce epithelial injury, fibrosis and irreversible tube scarring, ultimately compromising the reproductive function [47]. Fallopian tube organoids have emerged as physiologically relevant 3D models that faithfully preserve the cell diversity, epithelial polarity and secretory activity of the native tubal epithelium [27]. By closely reproducing the physiological microenvironment involved in gamete transport and fertilization, these models have become valuable tools to investigate epithelial homeostasis and the molecular mechanisms underlying tubal infertility. One of their first major applications has been the study of infection-induced tube damage. Chronic *C. trachomatis* infection has been successfully reproduced in human fallopian tube organoids generated from salpingectomy specimens. This allowed demonstrating that persistent bacterial infection induces profound epithelial remodeling, characterized by increased expression of stem cell-associated markers, including *LGR5*, *SOX9* and *NANOG*, enhanced epithelial proliferation and long-lasting epigenetic alterations of genes involved in cell differentiation and aging [38]. Importantly, this model showed that many of these molecular alterations persist after bacterial clearance, bringing new insights into the mechanisms of irreversible fibrosis and infertility. Subsequent studies confirmed the relevance of these organoids for investigating host–pathogen interactions and the cellular pathways involved in infection-associated infertility [40]. Moreover, fallopian tube organoids have recently expanded our understanding of the reproductive tract microbiota and its influence on tubal physiology. Using patient-derived organoids, Yu et al. [39] demonstrated that *Fannyhessea vaginae,* a species associated with bacterial vaginosis, induces a marked inflammatory response characterized by upregulation of genes encoding different cytokines and chemokines, particularly members of the CXCL family (Table 1). Conversely, exposure to the commensal bacterium *Lactobacillus crispatus* elicited minimal inflammatory activation and promoted immune-regulatory pathways, including TGF-β expression, supporting its protective role in epithelial homeostasis maintenance. These findings highlight the importance of the vaginal microbiota in reproductive health and establish fallopian tube organoids as robust experimental platforms for investigating host–microbiome interactions. Although their clinical applications remain at an earlier stage than those of ovarian organoids, fallopian tube organoids are increasingly recognized as versatile models to study epithelial regeneration, reproductive diseases and tubal infertility.

### 2.4. Endometrium and Endometrial Organoids

The endometrium is a highly dynamic tissue that undergoes cyclic remodeling under the coordinated actions of estrogen and progesterone to establish the receptive environment required for embryo implantation and pregnancy [48]. Its remarkable regenerative capacity relies on epithelial and stromal progenitor cells in the basal layer that regenerate the functional layer following each menstrual cycle. This cyclical remodeling depends on tightly regulated interactions of the epithelial, stromal, vascular, immune and endocrine compartments. Defects in endometrial regeneration, differentiation or receptivity are increasingly recognized as major contributors to female infertility and embryo implantation failure. Among the acquired uterine disorders, Asherman’s syndrome is characterized by intrauterine adhesions and fibrosis, resulting primarily from injury to the basal endometrium following uterine curettage or other intrauterine procedures [49]. Chronic genital infections, including genital tuberculosis, also may induce fibrotic remodeling of the endometrium [50]. Endometriosis is another major cause of female infertility. This chronic estrogen-dependent inflammatory disease concerns ~10% of women of reproductive age and is characterized by the growth of endometrial-like tissue outside the uterine cavity, leading to chronic inflammation, fibrosis and pelvic adhesions [51]. Endometriosis compromises fertility through multiple mechanisms, including altered tubal function, abnormal uterine contractility, progesterone resistance, defective endometrial receptivity and a persistent inflammatory microenvironment that impairs fertilization, embryo development and implantation.

Endometrial organoids have emerged as robust 3D models that faithfully reproduce the structural, functional and hormonal characteristics of the human endometrial epithelium [28,52]. By preserving the glandular architecture, epithelial polarity and hormone responsiveness, these models more accurately recapitulate the dynamic interactions between endometrial epithelial cells and their microenvironment compared with conventional 2D cell culture systems. Consequently, endometrial organoids have become valuable platforms to study endometrial homeostasis, embryo implantation and infertility-associated diseases. One of their major applications has been the development of regenerative approaches for Asherman’s syndrome. Human embryonic stem cell-derived endometrial organoids have successfully reproduced key features of the endometrial epithelium and demonstrated remarkable regenerative capacity following transplantation into experimental models of intrauterine adhesions. Organoid transplantation promoted endometrial repair by restoring the glandular architecture, enhancing tissue vascularization and significantly reducing fibrosis and intrauterine adhesions, highlighting their therapeutic potential for endometrial regeneration [41]. More recently, advances in tissue engineering, including the development of biomimetic extracellular matrices, natural and synthetic hydrogels, 3D scaffolds and microfluidic culture systems, have provided more physiologically relevant microenvironments that support organoid growth, differentiation and tissue organization, reinforcing their potential for regenerative medicine and fertility restoration [53]. Endometrial organoids are also powerful experimental models to study endometriosis. Patient-derived organoids generated from eutopic endometrium and ectopic endometriotic lesions faithfully preserve the histological architecture, molecular profile and hormonal responsiveness of the original tissues [43]. These models provide unique opportunities to investigate the mechanisms underlying lesion establishment, chronic inflammation, progesterone resistance and impaired endometrial receptivity (Table 1). They also represent valuable preclinical platforms for drug screening and for exploring candidate personalized therapeutic strategies (Figure 1).

Moreover, endometrial organoids are increasingly recognized as versatile platforms to study implantation biology, embryo–endometrium interactions and endometrial regeneration. Beyond disease modeling, female reproductive organoids also represent promising emerging platforms for reproductive toxicology, enabling the investigation of tissue-specific responses to environmental toxicants, endocrine-disrupting chemicals and pharmaceuticals across the ovary, fallopian tube and endometrium.

## 3. Male Genital Tract and Infertility

### 3.1. Overview

Male fertility depends on the coordinated function of the whole male reproductive tract (testes, epididymides, vasa deferentia, accessory sex glands and urethra) to ensure sperm production, maturation, transport and ejaculation. Testes are responsible for testosterone production and spermatogenesis to generate sufficient numbers of genetically and functionally competent spermatozoa. Following their release from the seminiferous tubules, immature spermatozoa migrate to the epididymis where they undergo structural, biochemical and functional maturation, acquiring motility and fertilizing capacity. Mature sperm is transported through the vas deferens and mixed with secretions from the seminal vesicles, prostate and bulbourethral glands that provide nutrients, buffering capacity and signaling molecules essential for sperm survival and function before ejaculation through the urethra [54]. As successful fertilization requires the coordinated function of each male reproductive tract component, abnormalities affecting any of these organs may compromise the reproductive potential. Indeed, male infertility is a multifactorial disorder resulting from defects in spermatogenesis, sperm maturation, sperm transport or ejaculation, as well as endocrine, genetic, infectious, anatomical, immunological and environmental factors. Non-obstructive azoospermia, oligozoospermia and asthenozoospermia are the most common clinical manifestations of impaired male fertility. Overall, male factors are involved in ~40% of infertility cases, either as isolated causes or in combination with female factors [55]. Despite significant advances in reproductive medicine, many forms of male infertility remain poorly understood because of the complexity of spermatogenesis and the lack of physiologically relevant experimental models that reproduce the highly organized architecture of the human testis and the interactions between germ cells and supporting somatic cells. These limitations have stimulated the development of advanced 3D models, particularly testicular organoids, that provide new opportunities to investigate testis development, spermatogenesis, male infertility and reproductive toxicology [29,56]. However, the technological maturity of male reproductive organoid models remains uneven across tissues. Current advances have primarily focused on testicular organoids, which are the most developed systems for studying testicular organization, spermatogenesis and reproductive toxicity. In contrast, organoid models of other components of the male reproductive tract, including the epididymis, vas deferens and accessory sex glands, remain comparatively less developed and have so far been only limitedly applied to human infertility research. Accordingly, the following section focuses mainly on testicular organoids, reflecting the current state of the field and the relative maturity of available models rather than the biological importance of the other male reproductive tract compartments.

### 3.2. Testis and Testicular Organoids

The testis is the primary male reproductive organ, with endocrine and exocrine functions through testosterone production and continuous sperm generation. Spermatogenesis begins at puberty in the seminiferous tubules and requires the coordinated interactions between germ cells, Sertoli cells, Leydig cells and the surrounding microenvironment. The complete differentiation of spermatogonia into mature spermatozoa takes ~74 days in humans [57]. Although spermatogenesis can persist into advanced age in some individuals [58], sperm concentration, motility and overall reproductive potential gradually decline with age [59]. Male fertility is also influenced by many environmental and pathological factors. Infectious diseases, particularly mumps orchitis, may result in irreversible germ cell depletion and azoospermia. Moreover, environmental exposures, including tobacco smoke [60], endocrine-disrupting chemicals and cannabis, are increasingly recognized as important contributors to male infertility by impairing spermatogenesis and sperm function. For instance, cannabis disrupts sperm ion-channel activity, thereby compromising sperm physiology [61]. Genetic abnormalities, including Y chromosome microdeletions, Klinefelter syndrome and pathogenic variants of genes involved in meiosis and spermatogenesis, are major causes of non-obstructive azoospermia and severe male infertility [62,63,64]. Testicular organoids have emerged as promising 3D models that recapitulate key aspects of testicular architecture, endocrine activity and germ cell–somatic cell interactions [29,56]. Compared with conventional 2D cell culture systems, these models better preserve the spatial organization of seminiferous tubules and provide physiologically relevant platforms to study spermatogenesis, endocrine regulation and male infertility. Murine testis organoids generated from prepubertal testicular cells successfully reproduce seminiferous tubule-like structures and maintain functional Leydig cells that produce testosterone. However, complete germ cell maturation into spermatozoa has not been achieved in vitro yet [65,66]. These findings demonstrate the capacity of organoid systems to reproduce important structural and functional features of the testis (Table 2).

**Figure 2:**
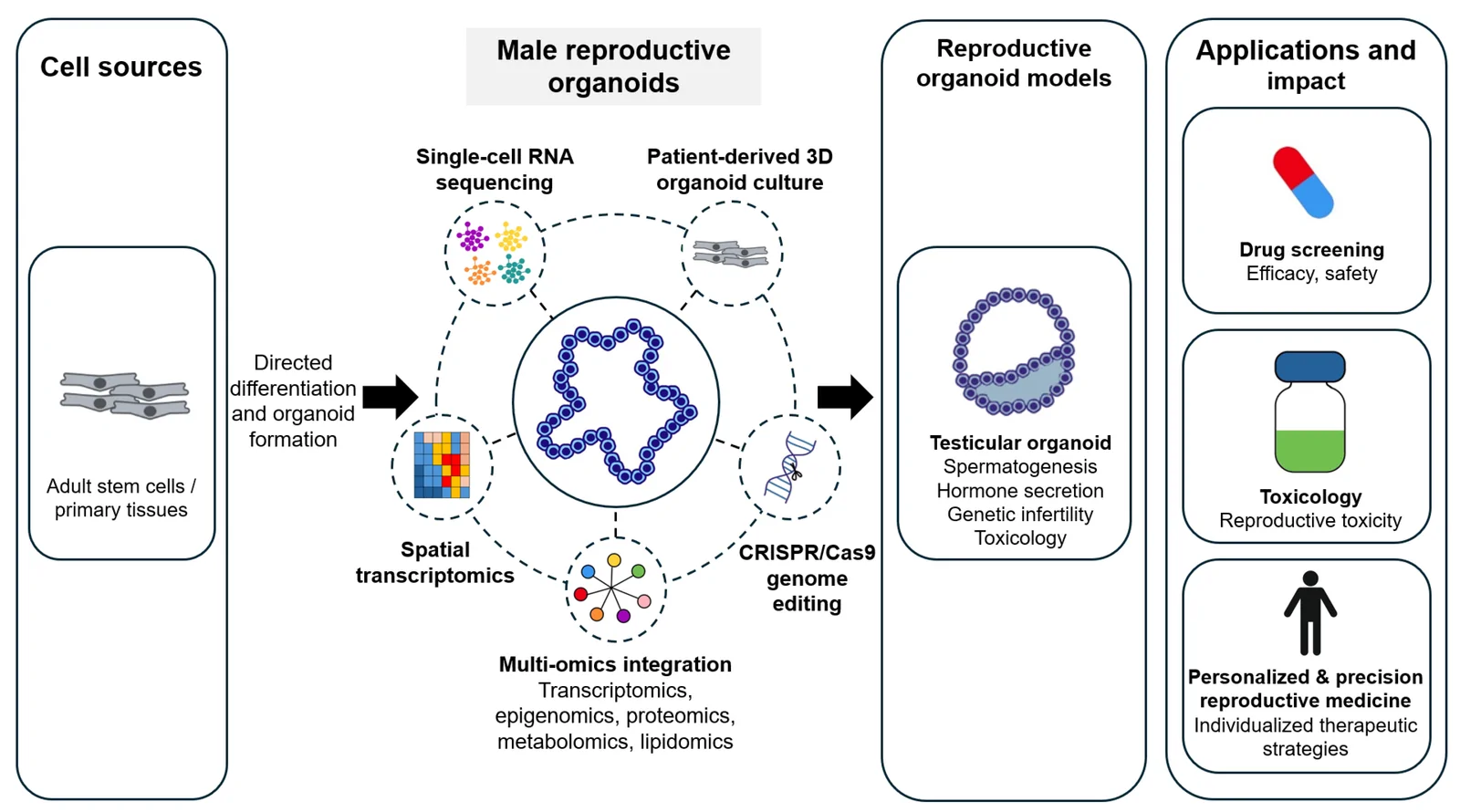
Next-generation male reproductive organoids that integrate genomics, multi-omics and bioengineering to study male infertility. Generation of testis organoids from adult stem cells and primary tissues combined with patient-derived cultures, single-cell transcriptomics, spatial transcriptomics, CRISPR/Cas9 and multi-omics technologies. These integrated models facilitate the study of spermatogenesis, endocrine function, genetic infertility, reproductive toxicology, therapeutic screening and precision reproductive medicine.

One of the most promising applications of testis organoids is for gonadotoxicity studies. Experimental models generated from rat [67] and porcine [68] testes have been successfully used to evaluate the effects of environmental toxicants on testis function. Exposure of rat-derived organoids to mono-(2-ethylhexyl) phthalate, a widely used plasticizer, induced marked disruption of Sertoli cell integrity, loss of spermatogonia and impairment of the blood–testis barrier through alterations in claudin-11 expression, reproducing key pathological features of toxicant-induced infertility [67]. Similarly, exposure to the antidepressant drug amitriptyline significantly reduced testis organoid viability and decreased the expression of *ZO-1*, *SOX9*, *STRA8* and *UCHL1*, indicating impaired Sertoli cell differentiation, disruption of the blood–testis barrier and defective germ cell development [69]. These studies illustrate the value of testis organoids to identify cellular targets of environmental and pharmacological toxicants and to provide mechanistic insights into male reproductive toxicity. Testis organoids also provide a promising framework for investigating the genetic basis of male infertility. In principle, the integration of patient-derived iPSCs with genome-editing technologies such as CRISPR/Cas9 could enable the modeling of genetic abnormalities affecting spermatogenesis, including pathogenic variants in meiotic genes such as *TEX11* and *SYCP3* [63,70]. However, compared with their established use in reproductive toxicology and studies of testicular organization, organoid-based modeling of specific human genetic causes of infertility remains at an early stage. Y-chromosome microdeletions, Klinefelter syndrome and specific infertility-associated gene variants should therefore be considered important targets for future organoid-based modeling rather than established applications. Such models could ultimately facilitate mechanistic studies and functional validation of candidate infertility genes (Figure 2).

## 4. Preimplantation Development, Implantation and Embryonic Organoids

Early human development begins immediately after fertilization, when the zygote undergoes a series of cleavage divisions followed by compaction to form the morula and subsequently the blastocyst between days 5 and 7. The blastocyst is composed of three distinct cell lineages that establish the foundation of embryonic and extraembryonic development: (i) the epiblast, located within the inner cell mass, gives rise to the embryo proper; (ii) the hypoblast contributes to the yolk sac; and (iii) the outer trophectoderm differentiates into the fetal component of the placenta, supporting implantation and maternal–fetal exchanges [71,72]. A successful pregnancy depends on the precise coordination between embryonic development and endometrial receptivity during the implantation window. Implantation is a highly regulated process that involves blastocyst apposition and adhesion, trophoblast invasion and the establishment of the early maternal–fetal communication. Defects affecting the embryo or the endometrium may compromise implantation and result in infertility or early pregnancy loss. On the maternal side, hormonal imbalance, immune dysregulation, inflammation and defective endometrial receptivity can impair implantation. In the embryo, chromosomal abnormalities, genetic defects and epigenetic alterations may compromise blastocyst development, cell differentiation and trophoblast invasion. Therefore, the precise temporal synchronization between embryo competence and endometrial receptivity represents one of the most critical determinants of reproductive success [73,74]. Despite its clinical importance, implantation remains one of the least understood stages of human reproduction. Ethical constraints on human embryo research and the limited availability of preimplantation embryos have considerably restricted mechanistic investigations. Conventional animal models only partially reproduce human embryogenesis because of significant species-specific differences in developmental timing, lineage specification and implantation mechanisms. These limitations have stimulated the development of embryonic organoids, particularly human blastoids, as physiologically relevant models to study early human development and embryo–endometrium interactions.

Human blastoids are stem cell-derived 3D structures that reproduce many structural, molecular and functional characteristics of human blastocysts. They contain the three principal blastocyst cell lineages (epiblast, hypoblast and trophectoderm) and constitute powerful experimental platforms to investigate lineage specification, early embryonic development and implantation biology [75,76,77]. Initial studies demonstrated that human pluripotent stem cells can self-organize into blastoid structures capable of reproducing the cellular composition of a blastocyst and of attaching to in vitro models of the human endometrium. They provided the first experimental system to investigate early embryo–maternal interactions in controlled conditions [77]. Afterwards, blastoid generation has been considerably improved by optimizing differentiation protocols and enabling large-scale production from naive human pluripotent stem cells [78]. More recently, refinements in stem cell reprogramming and 3D culture systems have expanded the developmental potential of human blastoids. Intermediate pluripotent states, generated during the transition from primed to naive iPSCs, have allowed the production of blastoids that reproduce the blastocyst organization and also developmental events close to the pre-gastrulation stage [79]. Moreover, chemically optimized protocols using naive pluripotent stem cells have been used to generate blastoids with DNA methylation landscapes that closely resemble those of natural human blastocysts and that progress toward molecular signatures characteristic of gastrulating embryos [80]. These advances have substantially improved the physiological relevance of embryonic organoids and broadened their applications for investigating the earliest stages of human embryo development (Table 3). Human blastoids have also emerged as promising platforms to study the genetic and epigenetic determinants of implantation failure, recurrent pregnancy loss and early embryonic disorders.

**Figure 3:**
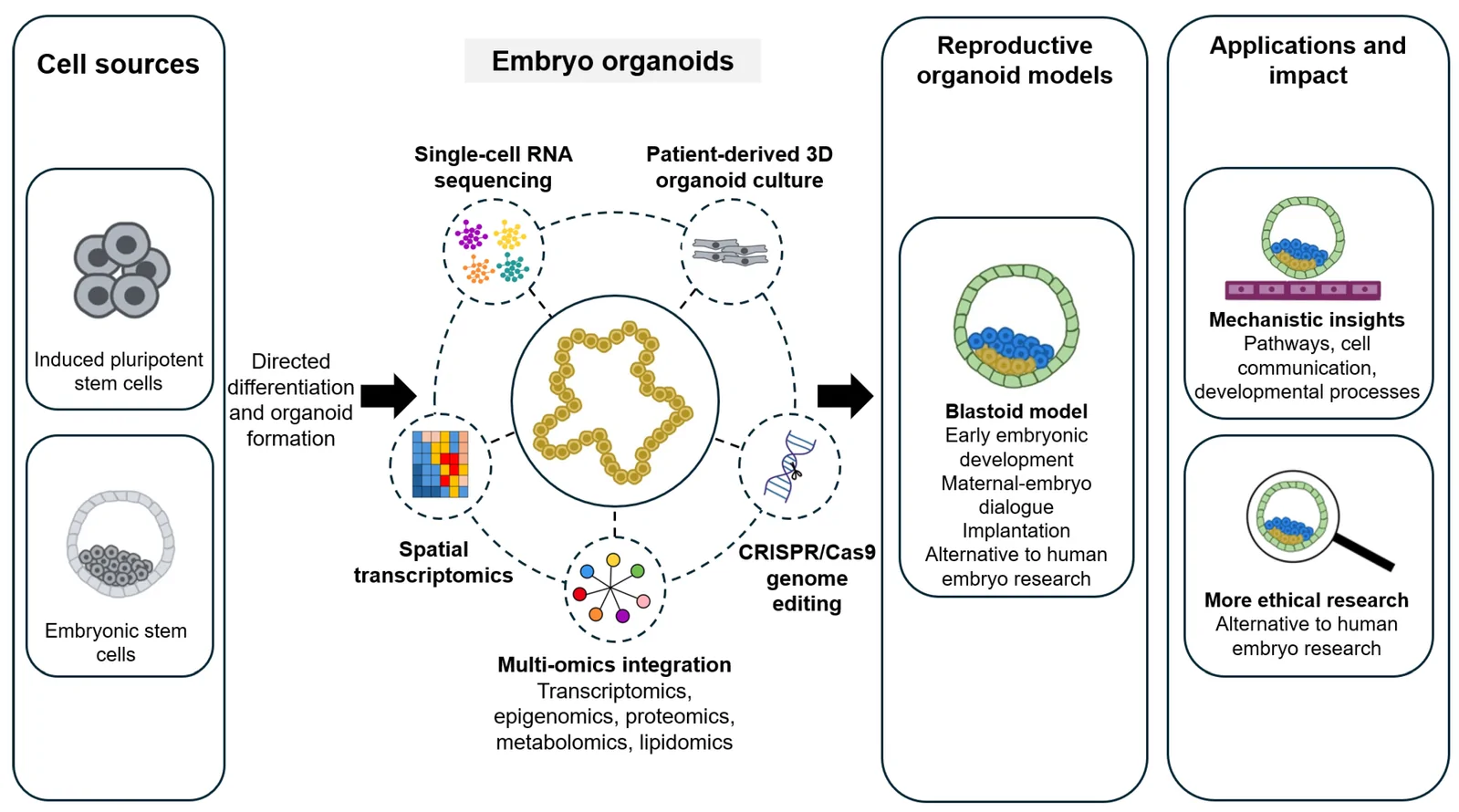
Next-generation embryo organoids that integrate genomics, multi-omics and bio-engineering to study early human development. Embryo organoids (blastoids) generated from pluripotent stem cells are integrated with single-cell transcriptomics, spatial transcriptomics, CRISPR/Cas9 genome editing and multi-omics. These models recapitulate key aspects of early embryonic development and implantation, providing mechanistic insights into developmental processes while offering an ethical alternative to the use of human embryos for research purposes.

Overall, reproductive organoids have fundamentally transformed infertility research by providing experimental systems that uniquely bridge human genetics, developmental biology and tissue physiology. Unlike conventional 2D cell culture systems, which fail to reproduce the structural and cellular complexity of reproductive tissues, and animal models, which often differ substantially from humans in reproductive development and endocrine regulation, organoids preserve the tissue-specific architecture, multicellular interactions and functional responses within a physiologically relevant human context. This unique combination allows the direct investigation of the cellular and molecular mechanisms underlying folliculogenesis, spermatogenesis, embryo implantation and early embryonic development and faithfully recapitulates disease-associated phenotypes. More importantly, reproductive organoids are uniquely suited to establish causal relationships between gene variants and reproductive defects. By integrating patient-derived stem cells, genome editing and advanced molecular profiling, these models can be used to functionally validate candidate infertility genes, reconstruct gene regulatory networks and characterize disease-associated cellular states that remain inaccessible in vivo. As a result, reproductive organoids are not simply advanced 3D culture systems, but have evolved into versatile experimental platforms to link genotype, epigenetic regulation, gene expression, cellular phenotype and tissue organization across the entire reproductive continuum (Figure 3). This conceptual transition provides the foundation for the future of reproductive research, in which the integration of multi-omics, computational biology and bioengineering will expand the mechanistic and translational potential of organoid-based models.

## 5. Emerging Multi-Omic Technologies Redefine Reproductive Organoids as Functional Genomic Platforms

Recent advances in genomics and systems biology are transforming reproductive organoids from conventional 3D tissue models into integrated platforms for functional genomics and precision reproductive medicine. Their combination with transcriptomics, epigenomics, genome engineering, spatial biology and computational approaches provides an unprecedented opportunity to connect genetic variation with molecular and cellular phenotypes in physiologically relevant human models (Figure 1, Figure 2 and Figure 3). Transcriptomic profiling remains central to organoid characterization. Bulk RNA sequencing provides global information on differentiation and tissue-specific molecular programs, whereas single-cell RNA sequencing resolves the cellular heterogeneity of reproductive tissues, identifies rare populations and reconstructs developmental trajectories involved in folliculogenesis, spermatogenesis, implantation and early embryogenesis [81]. Spatial transcriptomics complements these approaches by preserving tissue architecture and mapping cell-specific transcriptional programs and intercellular communication in their native spatial context. The combination of single-cell and spatial transcriptomics has already enabled the high-resolution characterization of the human ovary [82] and endometrial receptivity [83] and increasingly contributes to the study of spermatogenesis and male infertility [84]. Besides transcriptomics, integrative multi-omic approaches that combine genomics, transcriptomics, epigenomics, chromatin accessibility, proteomics and metabolomics provide a systems-level framework for reconstructing the molecular cascade linking gene variants to regulatory alterations and reproductive phenotypes. Such approaches are particularly relevant to infertility, where disease mechanisms are frequently linked to interactions between multiple molecular pathways rather than to isolated genetic defects. Single-cell multi-omics is already expanding the molecular characterization in reproductive medicine and early embryonic development [85] and has revealed disease-associated transcriptional and translational alterations in oocytes from patients with endometriosis [86]. A major advantage of integrating multi-omics and reproductive organoids is the possibility of moving from molecular associations to functional validation. The computational reconstruction of gene regulatory networks can help to identify transcription factors, regulatory elements and signaling pathways that regulate the differentiation and function of reproductive system cells. Moreover, patient-derived iPSCs can be genetically modified by CRISPR/Cas9 genome editing [87] before organoid generation to introduce or correct candidate pathogenic gene variants, generating isogenic models to directly establish genotype–phenotype relationships. This strategy positions reproductive organoids as powerful experimental platforms for validating infertility-associated genes and deciphering their underlying molecular mechanisms.

Organoid-on-chip technologies provide an additional level of physiological relevance by integrating reproductive organoids in microfluidic platforms to precisely control the cellular microenvironment, including dynamic nutrient and hormone delivery, fluid flow and interactions with stromal, vascular or immune components [88]. These systems can reproduce tissue–tissue interfaces and inter-organ communication that remain difficult to model in conventional static organoid cultures, as illustrated by microfluidic platforms that reproduce endocrine interactions in the female reproductive tract [89]. More recently, patient-derived endometrium-on-chip models that integrate endometrial epithelial organoids with stromal and endothelial compartments have enabled the reconstruction of endometrial architecture, vascular interactions and receptivity-associated features [90,91]. Importantly, coupling these micro-physiological systems with single-cell and multi-omic profiling provides an opportunity to dissect cell–cell communication and molecular responses in dynamically controlled conditions, thereby linking genomic alterations to functional tissue-level phenotypes [91]. Therefore, such platforms may bridge the gap between conventional organoid models and the complex physiology of the human reproductive system, while providing promising tools for mechanistic studies, drug testing and personalized reproductive medicine.

Artificial intelligence and machine-learning approaches further expand this framework by facilitating the integration of high-dimensional imaging data and single-cell, spatial-omic and multi-omic datasets. These methods can support automated cell classification, trajectory inference, spatial-domain identification, gene regulatory network reconstruction and biomarker discovery. The convergence of organoid technologies with artificial intelligence is increasingly being explored to improve disease modeling and predictive analysis and may ultimately support personalized therapeutic assessment, although such applications remain largely preclinical [92].

Collectively, the integration of reproductive organoids with single-cell and spatial omics, multi-omics, genome editing and artificial intelligence is establishing a new generation of functional genomic platforms to link genetic variation, molecular regulation, cellular phenotype and tissue organization. These approaches should accelerate the functional validation of infertility-associated genes, the identification of disease-specific biomarkers and therapeutic targets, and ultimately the development of precision reproductive medicine (Table 4).

## 6. Discussion

Reproductive organoids are reshaping human infertility research by providing experimentally accessible models that bridge genetic variation, molecular mechanisms and tissue-level phenotypes. Their major contribution extends beyond reproducing the architecture of reproductive tissues: they offer a human-specific framework in which the genetic, epigenetic and environmental determinants of infertility can be functionally investigated. This is particularly relevant because genomic discoveries have identified many infertility-associated gene variants, but establishing their biological consequences remains challenging. Patient-derived iPSCs combined with CRISPR/Cas9 genome editing are used to generate isogenic organoid models in which candidate gene variants are introduced or corrected, providing a powerful strategy to establish causal genotype–phenotype relationships [87]. Such approaches may facilitate the functional investigation of genes involved in folliculogenesis, meiosis and germ-cell development and help to move reproductive genetics from variant identification toward mechanistic understanding. Organoids also provide a unique opportunity to investigate gene–environment interactions, which are particularly relevant due to the multifactorial nature of infertility. Genetically defined reproductive tissues can be exposed to infectious agents, endocrine-disrupting chemicals, pharmaceuticals or inflammatory stimuli in order to monitor the molecular and functional consequences. For example, studies using fallopian tube organoids revealed persistent molecular and epigenetic alterations following *C. trachomatis* infection [38]. Similarly, testicular organoids have been used to investigate reproductive toxicity and the disruption of Sertoli and germ cell functions [67]. Extending reproductive toxicology to female organoid models represents an important future direction. Ovarian, fallopian tube and endometrial organoids could provide complementary human platforms to investigate tissue- and cell type-specific responses to environmental and pharmacological exposures and their consequences for folliculogenesis, endocrine function, tubal physiology and endometrial receptivity. Their integration with single-cell, spatial and multi-omic approaches could further elucidate the molecular pathways underlying female reproductive toxicity and improve the assessment of environmental and drug-related risks to fertility. More broadly, these integrated approaches enable the reconstruction of disease-associated cellular states and regulatory networks, providing a systems-level framework to connect genetic variation, molecular regulation and cellular dysfunction with infertility phenotypes.

Nevertheless, the current reproductive organoids remain simplified representations of the native tissues. Vascularization, innervation, immune and stromal components, extracellular matrix organization and systemic endocrine regulation are still incompletely reproduced. These limitations are particularly important in reproductive biology, where function depends on tightly coordinated multicellular interactions, including oocyte–granulosa communication, germ cell–Sertoli cell interactions and embryo–endometrial dialogue. Despite their advantages over conventional 2D cultures, reproductive organoids therefore do not fully reproduce the cellular complexity, tissue architecture and dynamic physiological environment of native reproductive organs. Incomplete maturation of some reproductive cell types and limited long-term phenotypic stability may further restrict their ability to recapitulate specific physiological and pathological processes in vitro.

While this review primarily focuses on testicular organoids, other components of the male reproductive tract, including the epididymis, vas deferens and accessory sex glands, are also essential for sperm maturation, transport and function. However, organoid models of these tissues remain comparatively less developed and their application to human infertility research is still limited, highlighting an important area for future investigation.

Therefore, research efforts should focus on restoring functionally relevant interactions rather than simply incorporating additional cell types. Bioengineering and organ-on-chip technologies offer promising solutions by introducing controlled perfusion, biomechanical cues, oxygen gradients and dynamic hormonal stimulation. In the future, interconnected reproductive micro-physiological systems could reproduce the communication between the ovarian, tubal, endometrial, embryonic, immune and vascular compartments, although the optimal level of complexity should be tailored to the biological question.

A major prerequisite for the broader implementation of reproductive organoids is improved reproducibility and standardization. Indeed, organoid phenotypes can vary depending on donor background, cell source, differentiation protocol, passage number, extracellular matrix and culture conditions, potentially affecting reproducibility across experiments and laboratories. Scalability and the lack of standardized culture and quality-control procedures represent additional challenges for the broader implementation of reproductive organoid models. Morphological similarity alone is not sufficient to establish physiological relevance. Standardized quality-control criteria should integrate molecular identity, epigenetic stability, cellular composition and tissue-specific functional endpoints. Benchmarking against primary human reproductive tissues using single-cell and spatial reference datasets will also be essential to determine reproductive organoid developmental maturity and physiological fidelity.

Beyond these experimental limitations, substantial barriers remain before reproductive organoids could be considered for integration into clinical practice. Their ability to predict individual reproductive outcomes or therapeutic responses has not yet been prospectively validated, and standardized criteria for model generation, characterization and functional assessment are still lacking. Moreover, patient-derived cells, pluripotent stem cells, genome editing and embryo-like models raise specific regulatory, ethical and biosafety considerations. Reproductive organoids should therefore currently be considered primarily as mechanistic and preclinical research platforms rather than validated tools for clinical decision-making. Further standardization, multicenter validation and demonstration of clinical predictive value will be required before their potential integration into precision reproductive medicine.

Embryo-like models require additional scientific and ethical considerations. Human blastoids provide unprecedented opportunities to investigate lineage specification, implantation and early developmental failure, but their increasing developmental fidelity raises questions about their biological equivalence to natural embryos and the appropriate ethical oversight [93,94]. Rigorous molecular and functional benchmarking and evolving regulatory frameworks will be essential as these models become increasingly sophisticated.

Overall, reproductive organoids should not be viewed as replacements for animal models, primary human tissues or clinical cohorts, but rather as complementary experimental platforms bridging genomic discovery and reproductive physiology. Their convergence with genome engineering, multi-omics, spatial biology, artificial intelligence and bioengineering provides new opportunities to establish mechanistic links between genetic variation and infertility phenotypes. Next-generation reproductive organoids with improved physiological fidelity, reproducibility and standardization could progressively move infertility research from descriptive associations toward mechanistic understanding and, ultimately, support the development of precision reproductive medicine.

## Figures and Tables

**Table 1:** Representative female reproductive organoid models and their applications in infertility research. Overview of representative ovarian, fallopian tube and endometrial organoid models, including their cellular origin, culture conditions and main experimental applications. The “Model maturity/principal limitation” column provides an indication of the current experimental maturity and/or main limitation of each model. Background shading is used to facilitate visual distinction between the different studies.

Tissue	Species	Cell Source	Organoid Medium	Model Maturity/Principal Limitation	Reference
Ovary	Human	hiPSCs	FSH, activin A, inhibin, BMP4, FGF2, EGF, LIF	Emerging/incomplete follicular maturation and limited reproduction of native ovarian complexity	Pierson Smela et al. 2023 [36]
	Rat	Granulosa cells collected by aspiration of antral follicles after ovarian dissection and theca cells isolated from tissues surrounding follicles	FSH, LH, estradiol, EGF, VEGF, insulin-transferrin-selenium	Proof-of-concept/non-human model and limited direct translation to human ovarian physiology	Sittadjody et al. 2017 [37]
Fallopian tube	Human	Anatomically normal fallopian tube tissue removed for benign diseases	Wnt3a, RSPO1, EGF, noggin, FGF10, nicotinamide, Y-27632, SB431542	Established epithelial model/limited stromal, immune, vascular and muscular components	Kessler et al. 2019 [38]
	Human	Anatomically normal fallopian tube tissue removed for benign diseases	Wnt3a, RSPO1, EGF, noggin, FGF10, nicotinamide, Y-27632, SB431542		Yu et al. 2024 [39]
	Human	Anatomically normal tissues removed during hysterectomies	Wnt3a, RSPO1, EGF, noggin, FGF10, nicotinamide, Y-27632, SB431542		Vollmuth et al. 2022 [40]
Endometrium	Human	Human embryonic stem cells	EGF, β-estradiol,	Relatively mature epithelial model/incomplete representation of stromal, vascular and immune compartments	Jiang et al. 2021 [41]
	Human	Endometrial tissue collected during diagnostic hysteroscopies	Y-27632, A83-01, nicotinamide, EGF, noggin, R-spondin-1, FGF-10, HGF		Hwang et al. 2024 [42]
	Human	Endometrium and ectopic lesions collected from women with endometriosis	β-estradiol, noggin, EGF, FGF2, FGF10, nicotinamide, R-spondin-1, A83-01, Y-27632, SB202190		Boretto et al. 2019 [43]

**Table 2:** Representative testicular organoid models and their culture conditions. Overview of representative testicular organoid models developed from different species, highlighting their cellular origin, culture conditions and current applications.

Tissue	Species	Cell Source	Organoid Medium	Model Maturity/Principal Limitation	Reference
Testis	Mouse	Testicles taken from 5- to 6-day-old mice	FSH, LH, retinol, EGF, FGF2, GDNF, *α*-MEM, dexamethasone	Preclinical/incomplete germ-cell maturation and species-specific differences	Richer et al. 2024 [65]
	Rat	Testicular cells from 4–5-day-old Sprague-Dawley rats	*α*-MEM, HGF, activin A, FSH, LH, testosterone, BMP-4, BMP-7	Preclinical & toxicology model/non-human and incomplete spermatogenesis	Sakib et al. 2022 [67]
	Pig	Testicular cells from 1-week-old Yorkshire-cross piglets	GDNF, FGF2, VEGF, SCF, retinol, hCG, FSH	Preclinical & toxicology model/non-human and limited validation of human infertility mechanisms	Cham et al. 2024 [68]

**Table 3:** Representative human blastoid models and their culture conditions. Summary of representative human blastoid models generated using pluripotent stem cell-based approaches, including the main culture conditions and references. These models provide experimental platforms to investigate early embryonic development, lineage specification and embryo–endometrium interactions. hESC (human embryonic stem cells), hiPSC (human induced pluripotent stem cells).

	Species	Cell Source	Organoid Medium	Model Maturity/Principal Limitation	Reference
Blastoid	Human	hESC + hiPSC	Lysophosphatidic acid (LPA), A83-01, PD0325901, LIF, Y-27632	Rapidly emerging/incomplete equivalence to natural blastocysts and important ethical and regulatory considerations	Kagawa H. et al. 2022 [77]
	Human	hESC + hiPSC	CEPT, A83-01, PD0325901, SB590885, WH-4-023, LPA		Yu et al. 2023 [78]
	Human	hESC	CEPT, A83-01, PD0325901, SB590885, WH-4-023, LPA		Tu et al. 2023 [79]
	Human	hESC + hiPSC	CEPT, LIF		Xie et al. 2025 [80]

**Table 4:** Emerging technologies integrated with reproductive organoids for infertility research. Overview of emerging technologies to be combined with reproductive organoid models, their main applications in infertility research and potential contributions to precision reproductive medicine.

Technology	Main Application	Infertility Application	Future Perspectives
Single-cell RNA-sequencing	Cell heterogeneity	POI, non-obstructive azoospermia, endometriosis	Biomarker discovery
Spatial transcriptomics	Cell-cell interactions	Implantation, folliculogenesis	Tissue architecture
CRISPR/Cas9	Functional genomics	Genetic infertility	Personalized medicine
Organoid-on-chip	Dynamic physiology	Fertilization, implantation	Drug screening
Multi-omics integration	Systems biology	Molecular pathways	Precision reproductive medicine

## Data Availability

No new data were created or analyzed in this study. Data sharing is not applicable to this article.
